# Selecting the Best Radiology Workflow Efficiency Applications

**DOI:** 10.1007/s10278-024-01146-2

**Published:** 2024-06-14

**Authors:** Prateek Bharadwaj, Michael Berger, Steven L. Blumer, Franziska Lobig

**Affiliations:** 1 Bayer AG, Berlin, Germany; 2 Simon-Kucher & Partners Strategy & Marketing Consultants GmbH, Bonn, Germany

**Keywords:** Radiology, Workflow efficiency, Workflow efficiency applications, Application assessment framework

## Abstract

In the rapidly evolving digital radiology landscape, a surge in solutions has emerged including more than 500 artificial intelligence applications that have received 510 k clearance by the FDA. Moreover, there is an extensive number of non-regulated applications, specifically designed to enhance workflow efficiency within radiology departments. These efficiency applications offer tremendous opportunities to resolve operational pain points and improve efficiency for radiology practices worldwide. However, selecting the most effective workflow efficiency applications presents a major challenge due to the multitude of available solutions and unclear evaluation criteria. In this article, we share our perspective on how to structure the broad field of workflow efficiency applications and how to objectively assess individual solutions. Along the different stages of the radiology workflow, we highlight 31 key operational pain points that radiology practices face and match them with features of workflow efficiency apps aiming to address them. A framework to guide practices in assessing and curating workflow efficiency applications is introduced, addressing key dimensions, including a solution’s pain point coverage, efficiency claim strength, evidence and credibility, ease of integration, and usability. We apply this framework in a large-scale analysis of workflow efficiency applications in the market, differentiating comprehensive workflow efficiency ecosystems seeking to address a multitude of pain points through a unified solution from workflow efficiency niche apps following a targeted approach to address individual pain points. Furthermore, we propose an approach to quantify the financial benefits generated by different types of applications that can be leveraged for return-on-investment calculations.

## Background

The field of radiology has seen a significant increase in digital solutions, with more than 500 artificial intelligence (AI) applications being 510 k approved for clinical use [1]. Additionally, there is a surge in digital solutions aiming to improve the workflow efficiency within radiology departments, namely, workflow efficiency applications (apps) [2]. The increasing demand for medical imaging—which in most places far exceeds capacity—coupled with a shortage of radiology staff poses a significant challenge to healthcare systems across the globe. This does not only result in a heavy workload for radiologists but also leads to longer waiting times for patients in need of imaging services [3]. Beyond this general challenge, a range of specific operational pain points exist along the various stages of the radiology workflow [4–6]. This creates opportunities for technology and informatics to generate meaningful improvements in the workflow, patient care, and staff satisfaction. Radiology workflow efficiency apps are becoming an increasingly relevant product category, aiming to streamline the radiology workflow by resolving operational pain points across different stages [7].

The landscape of workflow efficiency apps is diverse and can be categorized into distinct segments. Some apps are provided by manufacturers of imaging equipment to complement their hardware, while others are offered by Picture Archiving and Communication System (PACS) and Radiology Information System (RIS) vendors to enhance their existing software offerings. Apps from smaller, specialized companies add to this diversity. The solutions also vary in the scope of their offering. On the one hand, there are highly specialized apps that only address specific pain points along the workflow. On the other hand, there are comprehensive solutions covering the entire imaging workflow. While some apps focus solely on the radiology workflow, others extend their service to other departments within the hospital. Given the complex nature of the market, finding the best solutions among the numerous offerings poses a significant challenge to radiology providers. The apps’ diversity in origins, capabilities, and focus areas requires a careful evaluation to navigate this intricate landscape and to identify solutions that match the respective healthcare institutions’ requirements.

The current body of literature addressing radiology workflow efficiency is limited, with existing publications predominantly focusing on the general potential of artificial intelligence applications in tackling operational pain points in the radiology workflow [8, 9]. However, there is a gap with regard to the identification and selection of workflow efficiency apps in the market from the perspective of hospital and radiology practice decision-makers. Key questions surrounding the categorization of these apps, the criteria for an objective assessment of their value, their suitability for different institutional healthcare settings, and the quantification of their economic potential remain largely unexplored.

This paper seeks to address this gap in the literature and shed light on how to identify and select the best radiology workflow efficiency applications. It aims to provide a guide by introducing key criteria essential for assessing these apps, incorporating them into a structured assessment framework that facilitates an objective evaluation and prioritization of available apps. Additionally, it introduces an approach to quantifying the economic benefits derived from the implementation of different workflow efficiency apps.

## Methods

The concepts presented in this article draw upon the extensive experience and expertise in the radiology domain of Bayer and the management consultancy Simon-Kucher. These perspectives are further complemented by desk research and the insights from qualitative expert interviews. We conducted a targeted literature review of previous investigations of the radiology workflow, associated pain points, and the role of workflow efficiency apps. Additionally, we gained the perspective of a total of 31 key decision-making stakeholders, including radiology department heads, as well as hospital finance and IT specialists across institutions in the United States, the UK, and Germany. All interviewed experts were highly familiar with the radiology workflow, had at least 5 years of experience in their role, and had personally assessed or used workflow efficiency apps in this context as part of their role. Their views and assessments were collected in the form of 60-min in-depth virtual interviews, including both open-ended questions and closed-ended questions requesting ratings on pre-defined scales in conjunction with a rationale for the rating.

We followed a five-step approach to derive our concepts. First, we mapped the radiology workflow to understand and classify operational pain points along the different workflow steps according to their level of priority. The level of priority was assessed based on the perceived impact and frequency of the identified pain points. Perceived impact focuses on the severity of a pain point’s effect along relevant dimensions whenever it occurs. These dimensions include patient well-being but also level of potential time loss, which can impact staff satisfaction and hospital financials. Similarly, the perceived frequency assesses the regularity of occurrence. Some pain points occur in all or most radiology departments on a patient-by-patient basis, whereas others only affect a small share of patients in a subset of hospitals. Second, we identified available workflow efficiency apps through a large-scale screening before applying several exclusion criteria to create a shortlist of relevant workflow efficiency apps. Third, we categorized the shortlisted apps by their features to get an understanding of the functionalities critical to addressing key pain points. Fourth, we conducted a thorough evaluation of potential criteria to evaluate workflow efficiency apps and built an assessment framework around them that was subsequently applied on the shortlisted apps. Lastly, we developed an approach to approximate the financial benefits of different types of workflow efficiency apps as this is the key decision-driver of economic stakeholders within hospitals, who can act as gatekeepers to their adoption and implementation.

### Workflow and Pain Point Mapping

**Fig. 1:**
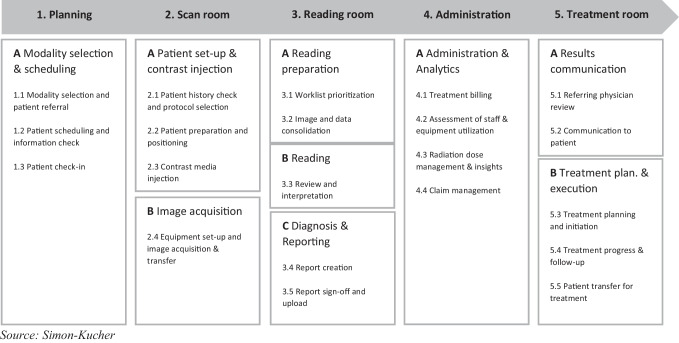
Radiology workflow divided into its substages and key activities

**Fig. 2:**
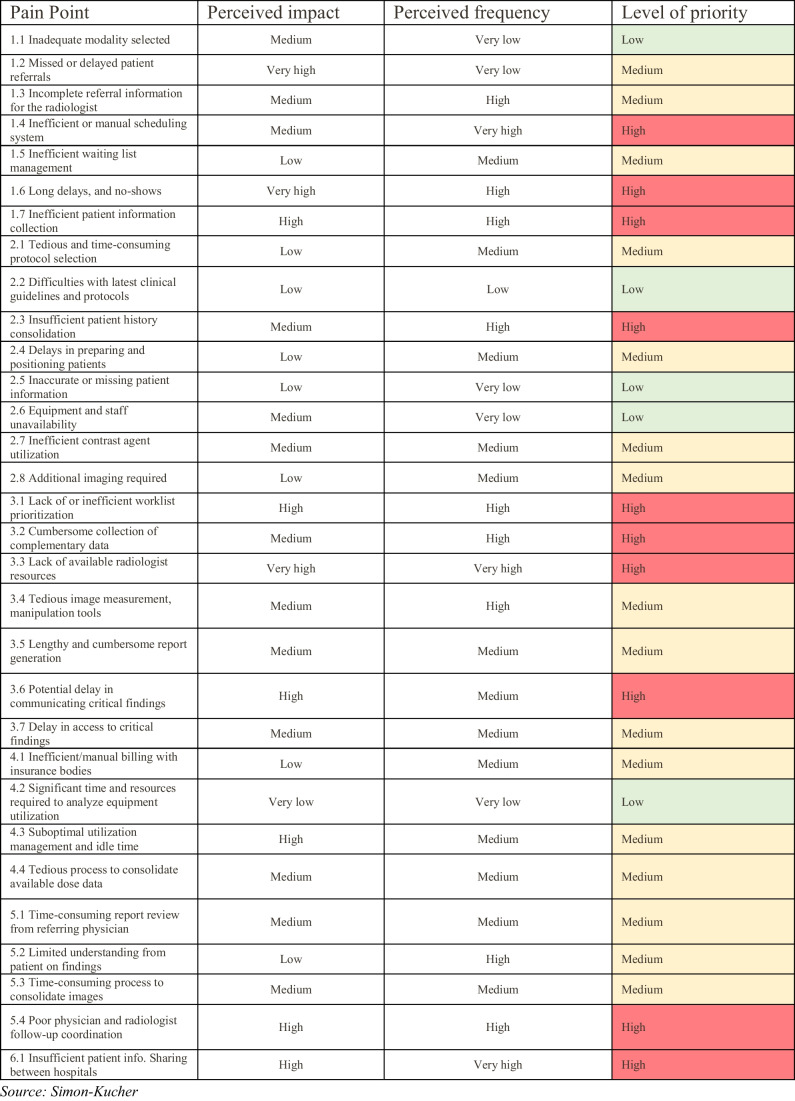
Classification of pain points into low, medium, and high priority pain points

As a starting point, we mapped the radiology workflow, identifying five main stages: (1) planning, (2) scan room, (3) reading room, (4) administration, and (5) treatment room. For each of these stages, substages and key activities were defined (Fig. 1). Subsequently, we identified 31 relevant pain points embedded within the current radiology workflow across their substages and key activities. Pain points were defined and subsequently prioritized based on findings from the initial literature review and knowledge of the authors and complemented by insights derived from a workshop with eight associated industry experts as well as the 31 external interviews conducted with experienced hospital decision-makers. The prioritization of pain points was based on the two criteria of perceived frequency and perceived impact. In both the internal workshop and the external expert interviews, the ratings were gathered via two 5-point scales ranging from 1 (“almost never” for frequency and “insignificant” for impact) to 5 (“very often for frequency” and “major” for impact). There was a very high level of congruence between the internal and external perspective. In cases of discrepancies, the perspective of external experts took precedence. Based on this assessment, we classified the pain points into three different levels of priority. Specifically, 10 pain points were defined as “high priority,” 16 as “medium priority,” and 5 as “low priority” (Fig. 2).

### App Identification and Filtering

In large-scale online research, we systematically identified relevant workflow efficiency applications, leveraging a broad range of sources such as the American College of Radiology Data Science Institute Database, Pitchbook, AngelList, and Crunchbase. This research allowed us to compile an initial list of potential candidates. To screen out less relevant apps from this longlist, several exclusion criteria were applied, including the following:

- Absence of publicly available information
- Primary focus on clinical claims (i.e., support in image interpretation)
- Replacement of existing IT infrastructure (e.g., PACS, RIS, or EMR solutions)
- Lack of specific focus on radiology
- Lack of testimonials or case studies validating implementation in at least one institution

PACS, RIS, and EMR solutions were excluded from the assessment. For all shortlisted apps passing this assessment, we mapped the high- and medium-priority features they address through their offering. In preparation for thoroughly evaluating the apps using the assessment framework at a later stage, we excluded all apps that did not address at least one pain point categorized as “high priority.” This selection process ensures a focused and meaningful shortlist of workflow efficiency apps as basis for a detailed assessment, in line with the most critical unmet needs in the context of radiology workflow efficiency.

### App Categorization by Features

**Fig. 3:**
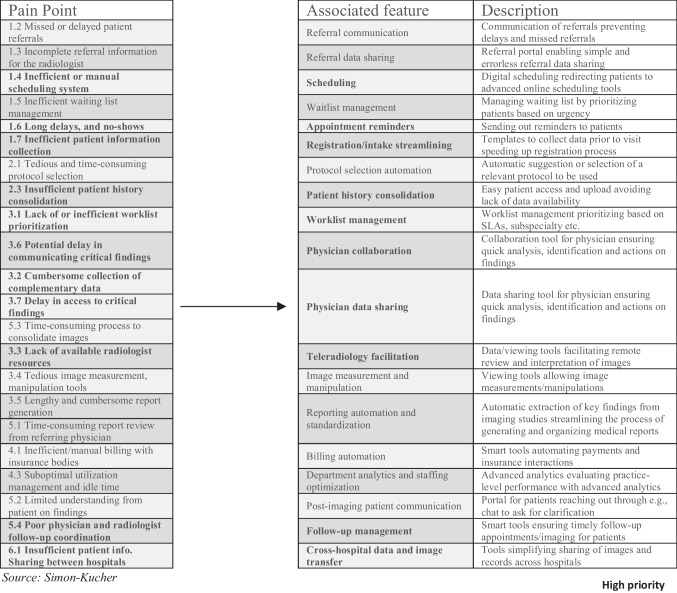
Allocation of medium and high priority pain points to the most relevant app features

Building upon an analysis of the identified pain points and shortlisted workflow efficiency apps, we defined distinct features inherent to these apps. Through a systematic process of assigning medium and high priority pain points to features of workflow efficiency apps, a set of 19 features was derived. This step resulted in the definition of 10 features categorized as “high priority” and 9 features as “medium priority” (Fig. 3). This categorization not only provides a nuanced understanding of the functionalities critical to addressing key pain points but also serves as a valuable criterion for the subsequent assessment and comparison of workflow efficiency apps in the radiology domain.

### Assessment Framework

In the subsequent step, we conducted a thorough evaluation of shortlisted workflow efficiency apps along an assessment framework encompassing five key criteria: (1) pain point coverage, (2) efficiency claim strength, (3) evidence/credibility, (4) ease of integration, and (5) usability.

Pain Point Coverage. An integral aspect of evaluating workflow efficiency apps lies in their ability to address relevant pain points through their set of features. An appropriate initial step to any assessment of these apps is to systematically evaluate which specific pain points are addressed by a solution. This process ensures a comprehensive understanding of how well the respective solution can address existing inefficiencies along the radiology workflow.

Efficiency Claim Strength. A critical aspect in evaluating workflow efficiency apps is assessing the strength of the efficiency claims made by the app providers. While all app manufacturers commonly assert the efficiency improvements their apps can deliver, these claims can be assessed in more detail by objectively judging their level of tangibility. Given that efficiency claims and ROI calculators may typically not be taken at face value by relevant decision-making stakeholders, it is recommended to consider them merely as initial evaluation points. In particular, when multiple apps provide the same feature, and manufacturers present varied claims, a critical assessment of the claims’ credibility and relevance is crucial, which is therefore accounted for in the following criterion.

Evidence/Credibility. In the evaluation of workflow efficiency apps, an objective review of the existing evidence and credibility assumes significant importance. Since efficiency claims are rarely proven by scientific evidence, the availability of successful case studies and testimonials becomes a critical dimension for consideration. Ideally, a solution should have demonstrated successful implementation in radiology institutions, garnering positive feedback and thereby establishing a reputable brand in the market. In addition, it can be highly valuable to engage with radiologists and institutions that have already implemented the app to understand the implementation success in more detail. This may involve direct communication, site visits, or the sharing of firsthand experiences, especially for substantial investments.

Ease of Integration. The ability of a solution to seamlessly integrate into different baseline hospital IT infrastructure is pivotal. If such integration is unattainable, a solution will most likely not be implemented. Considering that hospitals are often equipped with robust PACS and RIS systems, a workflow efficiency app must seamlessly integrate with these existing systems, as hospitals are reluctant to change PACS or RIS providers but are more willing to adopt a different workflow solution that is compatible with their current IT infrastructure. Engaging technical and IT stakeholders early in the evaluation process is considered best practice to assess the feasibility of a potential integration of the workflow app into the existing IT ecosystem.

Usability. In instances where various solutions exhibit comparable performance across objective criteria, usability can ultimately become a decisive factor. While its evaluation is best conducted subjectively by users in the context of a demo or even a pilot, its relevance should not be underestimated. An app that proves difficult to use may necessitate extensive staff training and face resistance from internal stakeholders, potentially resulting in low usage. Conversely, an app which is easy to use can ensure fast acceptance and widespread adoption within an institution. Recognizing this impact of usability on user engagement and satisfaction is crucial for optimizing the successful integration and utilization of workflow efficiency apps.

**Fig. 4:**
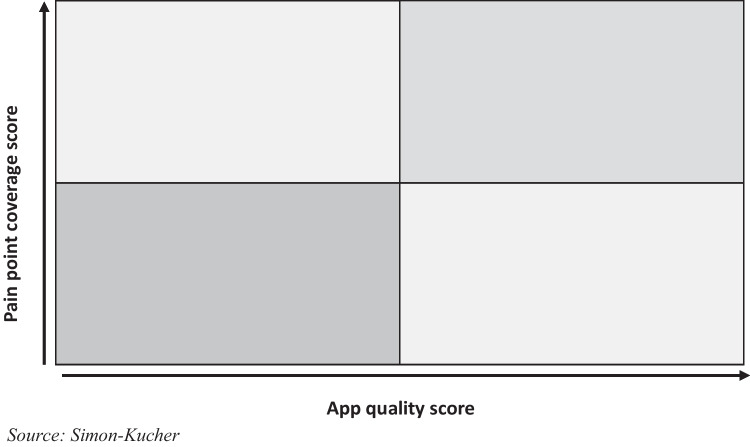
Two-dimensional workflow efficiency application assessment framework

Our final assessment framework was designed as a two-dimensional matrix, illustrating “pain point coverage score” on the y-axis and the “app quality score” on the x-axis, which is determined by evaluating efficiency claim strength and evidence/credibility (Fig. 4). More technical aspects related to ease of integration and usability were excluded from the assessment due to the need for individualized evaluation and the ambition to limit the complexity of this framework for a first line evaluation of apps.

**Fig. 5:**
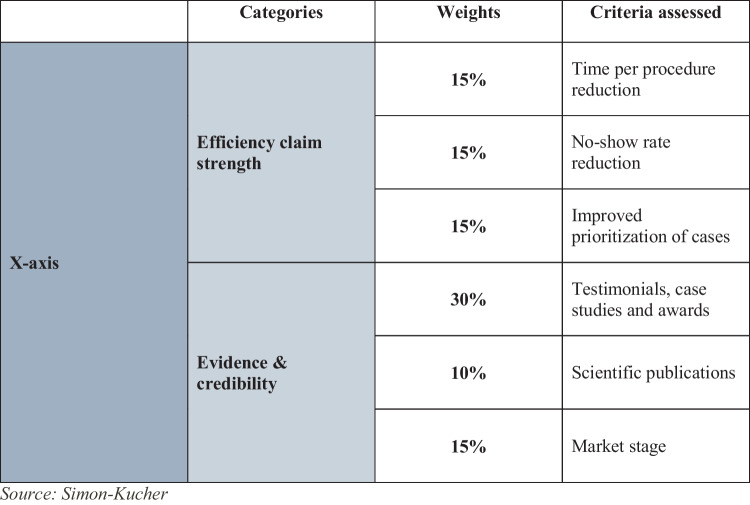
App quality score evaluation framework

To derive one overall app quality score per assessed app (x-axis), we developed a systematic assessment methodology with the components of efficiency claim strength contributing 45% and the evidence/credibility 55% of the weight to the overall score, respectively. The decision to assign 45% weight to efficiency claim strength and 55% weight to evidence/credibility was based on a comprehensive analysis of the impact of these components on app quality. Efficiency claim strength was deemed crucial in assessing the practical utility of the app, while evidence/credibility was given greater weight to emphasize the significance of robust evidence in app evaluation. Each component consisted of different criteria and objective 5-point evaluation scales were developed for the assessment of each criterion. The strength of efficiency claims provided was assessed across three criteria, reflecting different types of claims that the value proposition of a workflow efficiency app may entail: time per procedure reduction, no-show rate reduction, and improved prioritization of cases, each contributing 15%. We included all three and weighted all three criteria equally to acknowledge the benefit of workflow efficiency apps able to address multiple of them within a single solution, conscious of the fact that many are only designed to address one of the three. Similarly, evidence/credibility was evaluated through three criteria as well: testimonials, case studies and awards with the highest weight of 30%, scientific (peer-reviewed) publications with 10%, and market stage with 15% weight (Fig. 5). While we acknowledge that not many applications have robust evidence, the assignment of 10% to peer-reviewed publications was made with the understanding that while not all apps may have peer-reviewed publications, those that do should receive appropriate recognition for their evidence-based support. The evaluation scales were designed to ensure neutrality and scalability in the assessment of different types of workflow efficiency apps purely based on publicly available data obtainable through secondary research. For instance, the three criteria within the efficiency claim strength were assessed individually with the following 5-point scale:

1. A claim is made without any quantification.
2. A claim is made that gives an approximate quantification, as in e.g., “significant.”
3. A quantified claim is made that shows limited performance.
4. A quantified claim is made that shows medium performance.
5. A quantified claim is made that shows strong performance OR the app manufacturer proactively provides a tool to calculate potential financial benefits achieved with this efficiency driver.

For the calculation of the pain point coverage score (y-axis), a multiplier was assigned to each pain point based on its level of priority determined in the initial step of the work. High priority pain points received a multiplier of three, medium priority pain points were assigned a multiplier of one, and low priority pain points were disregarded with a multiplier of zero. This approach allows for a transparent scoring system, in which workflow efficiency apps could theoretically achieve a maximum pain point coverage score of 45 if a solution addressed all identified medium and high priority pain points within the radiology workflow. By calculating the weighted sum, both the quantity and the relevance of the pain points addressed by an app were considered in the pain point coverage score.

Applying this two-dimensional assessment framework can facilitate a fair and transparent evaluation of workflow efficiency apps based on their app quality and pain point coverage scores. The objectiveness of the framework and the fact that it solely leverages publicly available information allows for scalability. As the market develops rapidly, newly emerging apps can be added to the assessment and the scores of existing apps can easily be adjusted when additional information about them becomes available.

The actual assessment of all workflow efficiency apps on the final shortlist applying this assessment framework was conducted by the authors. We leveraged information available on the website of the app providers and additional information provided by the app providers upon request. Individual debatable cases were discussed in a larger group within the team to come to a consensus decision.

### ROI Quantification

Workflow efficiency apps can yield a broad range of efficiency gains and their impact can be measured in various ways. Many apps enhancing the workflow may enhance patient care and staff satisfaction leading to a reduced incidence of burnout among radiologists and other staff within the radiology department. However, the ability to show the direct economic impact of an app on hospital financials in the form of a compelling business case is typically demanded by economic stakeholders as institutions operate under limited budgets. Thus, we decided to focus our investigation on efficiency gains that have a direct impact on hospitals’ financials, either by increasing revenues and or decreasing operating costs. However, it must be noted that the exact impact of workflow efficiency apps on hospitals’ financials heavily depends on the funding system the hospital operates in. While some app manufacturers even provide concrete economic value claims or ROI calculators for their respective solutions on their website, analyses by app manufacturers are often perceived with skepticism by hospital decision-makers aiming to curate workflow efficiency apps. As part of our work, we therefore aimed to develop an approach to approximate the financial benefits associated with distinct features of workflow efficiency apps.

## Results

**Fig. 6:**
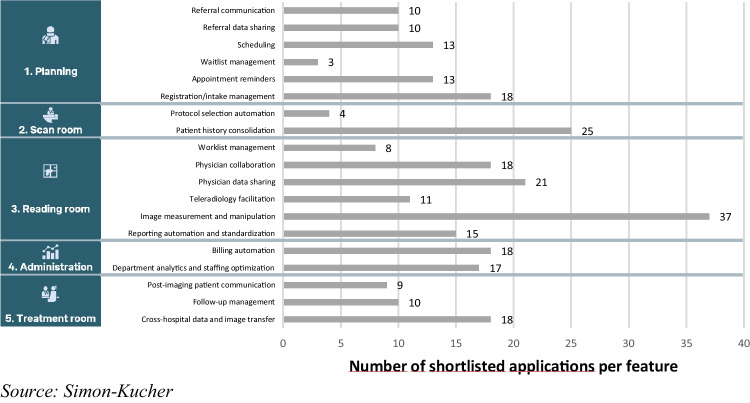
Number of assessed workflow efficiency applications per feature

The initial longlist of potential apps for assessment comprised a total of 891 candidates. Through careful screening, 89 apps were shortlisted and categorized based on the features they cover, while all others were excluded due to meeting one or multiple of the defined exclusion criteria. This approach enables the development of a systematic overview of the number of workflow efficiency apps covering each of the defined features (Fig. 6). Finally, additional 43 apps were excluded as they do not address a pain point labeled as “high priority.” This resulted in a final selection of 46 apps for in-depth assessment.

**Fig. 7:**
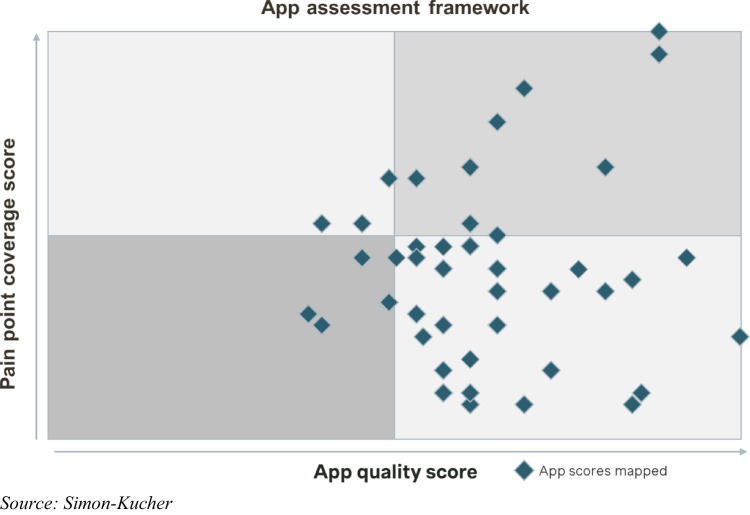
Workflow efficiency application assessment framework featuring 46 workflow efficiency apps

**Fig. 8:**
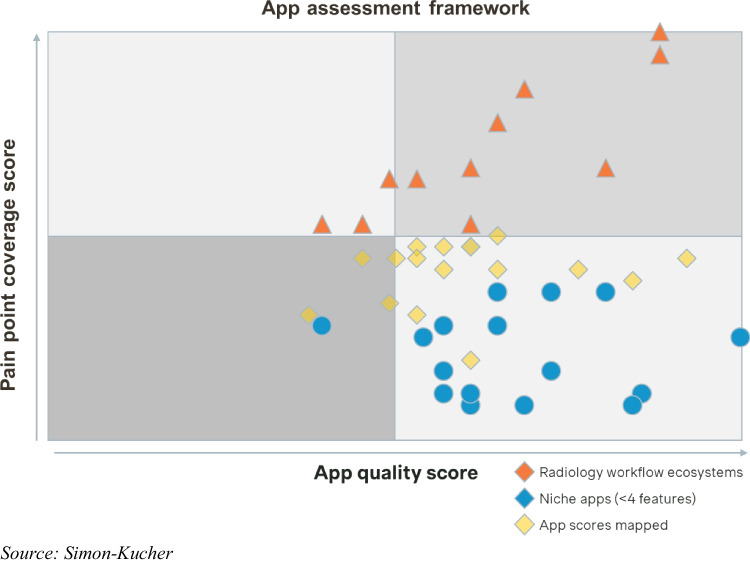
Workflow efficiency application assessment framework featuring 46 workflow efficiency apps, categorized into ecosystems and niche apps

The result of our assessment framework provides a visual representation of all assessed workflow efficiency apps, highlighting their performance along the two dimensions of “pain point coverage score” and “app quality score.” The resulting scatterplot features 46 apps addressing at least one high-priority pain point (Fig. 7). Upon analyzing the plotted workflow efficiency apps in the assessment framework, a clear distinction emerges: some solutions comprehensively cover most of the defined features, while others have a clear focus on single or only a limited set of features. This necessitates a categorization into workflow efficiency ecosystems and workflow efficiency niche apps, with a third category for those that do not fall into either group (Fig. 8). A workflow efficiency ecosystem incorporates a broad range of features aiming to address all critical pain points along the radiology workflow with a single solution or different modular solutions by the same manufacturer. Based on their high pain point coverage score, 11 applications were defined as workflow efficiency ecosystem. On the other hand of the spectrum, a workflow efficiency niche app incorporates a limited set of features and tends to excel in a targeted approach to particular pain points only. Leveraging this framework enables a prioritization strategy, emphasizing top-performing ecosystems in the top right quadrant and top-performing niche apps in the bottom right quadrant, facilitating informed decision-making based on both an app’s pain point coverage and app quality score.

**Fig. 9:**
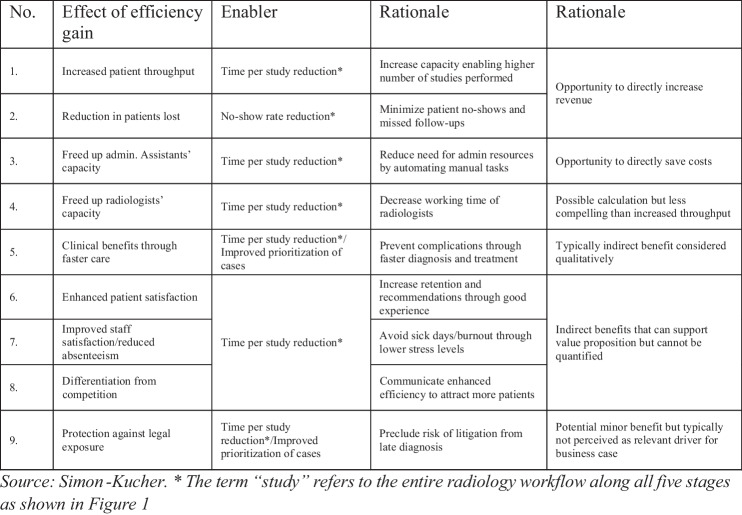
Overview of different effects of efficiency gains with its respective enabler

In addition to the criteria assessed in the assessment framework, quantifying the direct financial benefit of top-performing workflow efficiency ecosystems and niche apps, guided by our assessment framework, can aid decision-makers in selecting the most suitable apps for further individual evaluation. The direct financial benefit is apparent for features that can directly increase revenue or save costs through one of three key dimensions of efficiency gains. These dimensions include the following:

1. Increased patient throughput, leading to revenue increases due to a higher number of imaging studies performed within a given timeframe
2. A reduction in patient no-shows, contributing to revenue increases due to a higher number of imaging studies performed within a given timeframe
3. Freed up administrative assistants’ capacity, resulting in tangible cost savings (Fig. 9)

**Fig. 10:**
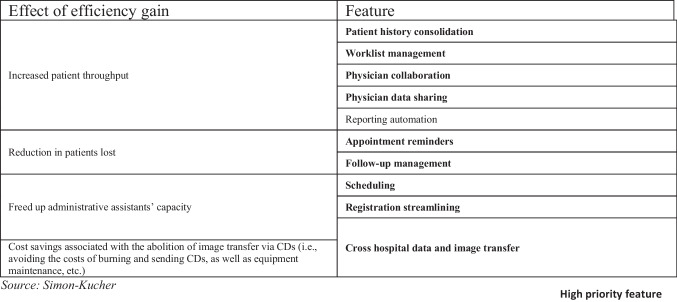
Allocation of the prioritized features to their respective effect of efficiency gain to allow for financial benefit quantification

**Fig. 11:**
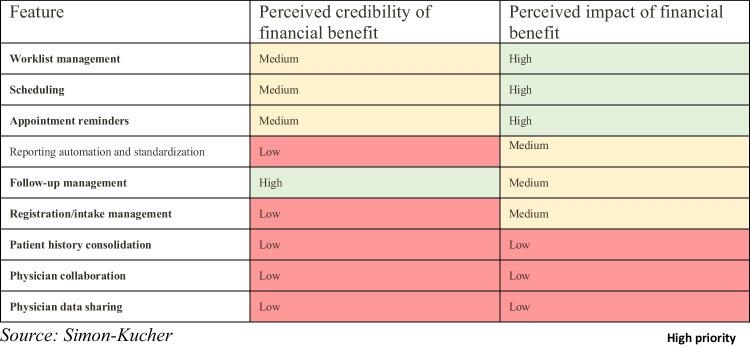
List of direct ROI generating features ranked by its order of magnitude

For instance, the financial benefit of an AI-based follow-up management app can be approximated by a reduction in the share of patients lost as they do not perform the required follow-up imaging (Fig. 10), thereby increasing the total number of imaging studies performed and directly impacting revenue. However, the perceived credibility and impact of financial benefits vary among features, as ascertained in interviews with decision-making experts (Fig. 11). This emphasizes the importance of considering the prioritization of apps that incorporate features with credible and impactful financial benefits.

## Discussion

The emerging market for workflow efficiency apps distinct from the more established field of clinical decision-support apps is highly complex. Many solutions with a broad range of use cases have been launched by a diverse set of providers, ranging from established corporations in the field of radiology to small startup companies. Hence, a thorough screening and evaluation of available solutions is required. With this analysis, we embark on an initial assessment of this market and provide a structured overview of the diverse field of workflow efficiency apps along a clearly defined set of dimensions. As a limitation, it should be noted that our analysis focused on workflow efficiency apps that are not incorporated into PACS, RIS, and EMR solutions. However, to obtain a comprehensive understanding of workflow efficiency solutions in radiology, these systems should also be considered. Furthermore, there is no objective metric or gold standard to benchmark the results of this analysis. Nevertheless, it should prove as a helpful springboard for individual institutions looking to discuss and subsequently address inefficiencies in their radiology workflow through workflow efficiency apps.

A categorization of distinct types of workflow efficiency apps is essential, with some solutions offering comprehensive coverage of defined features, while others focus on a more limited set, resulting in the differentiation of workflow efficiency ecosystems and workflow efficiency niche apps. These archetypes cater to different needs, prompting a discussion on the specific situations in which each type of app is beneficial for which type of institution. Workflow efficiency ecosystems, with their broad range of features and seamless integration, are particularly suitable for institutions dealing with a multitude of pain points arising from error-prone and/or inefficient existing IT infrastructure and larger healthcare networks aiming for standardization of systems and software across their institutions, resulting in the need for an end-to-end workflow solution to fix these issues. Conversely, workflow efficiency niche apps, excelling in targeted approaches to specific pain points, are suited for any institution aiming to address inefficiencies in the radiology workflow. However, their value proposition may be more attractive to institutions that are not looking for a workflow efficiency ecosystem as their radiology department may already be equipped with a relatively efficient, well-functioning radiology IT infrastructure provided by their RIS, PACS, EMR, or OEM provider. The applications may also appeal to those institutions facing budget constraints and/or having limited interest in enhancing digitalization of still largely manual and analog workflows.

While the framework covers a broad range of aspects, including the breadth and priority level of pain points covered by an app as well as its efficiency claim strength and evidence and credibility, it should only be viewed as a preliminary guide. More technical considerations like the ease of integration and usability were excluded from the analysis and it must be noted that first-hand usage in clinical practice was not a part of the assessment. Furthermore, the exact pain points along the radiology workflow and their level of priority were defined on an overarching level, even though they certainly differ from one institution to another. Individual institutions should tailor the assessment to their respective situation and needs. Moreover, it is advisable to pilot preferred apps to gain firsthand experience for a more comprehensive evaluation of their actual impact on clinical practice.

Similarly, the developed approach towards quantifying the financial benefits of workflow efficiency apps can serve as valuable starting point for further research in this field. Yet, the impact of implementing an individual app within the unique context of an individual institution necessitates an additional layer of detail in the calculation.

As navigating the opaque landscape of workflow efficiency apps is a complex endeavor for institutions looking to resolve their operational pain points and boost efficiency in the radiology department, assessing and curating solutions internally may not be a feasible and effective approach. The process of identifying, categorizing, and objectively assessing the performance of different apps prior to running internal pilots may sometimes exceed the internal resource capacities and capabilities of institutions. Therefore, in these situations, making use of platforms featuring a suite of curated apps by third-party companies can save resources and ensure that top-performing apps are selected based on an evaluation by experts in the field.

## Conclusion

The realm of workflow efficiency apps presents significant potential to enhance the radiology space, especially against the background of a steady increase in demand for medical imaging. Yet, the landscape of apps available is diverse and opaque. Given this situation, our work proposes a systematic approach to organize the market landscape by associating and aligning features of workflow efficiency apps with existing pain points in the radiology workflow. This categorization differentiates between comprehensive workflow ecosystems, targeting a multitude of pain points, and niche apps, focusing on individual pain points only. Upon an assessment of potential criteria for assessment of an app’s quality, we focused on an evaluation of the efficiency claims strength and an examination of the credibility of these claims based on the evidence available to support them in the form of testimonials and case studies. In line with the objective of our study, the developed assessment framework allows to prioritize workflow efficiency apps in the top right (ecosystems) and bottom right (niche apps) quadrant for further individual evaluation.

This approach can serve as valuable starting point for institutions aiming to explore the market of workflow efficiency apps and to identify the best one for their unique situation. However, as the detailed evaluation requires significant time and resources, entering into an agreement with a curated marketplace of workflow efficiency apps can present a viable alternative to tackling this complex topic in an isolated approach.

Moreover, this work provides an approach to conduct calculations of the financial benefit of features contributing either to revenue growth through an increased number of studies performed within a specific timeframe or cost savings derived from freed up administrative assistants’ capacity.
